# Relationship between Physical Demands and Player Performance in Professional Female Basketball Players Using Inertial Movement Units

**DOI:** 10.3390/s24196365

**Published:** 2024-09-30

**Authors:** Javier Espasa-Labrador, Carlos Martínez-Rubio, José María Oliva-Lozano, Julio Calleja-González, Marta Carrasco-Marginet, Azahara Fort-Vanmeerhaeghe

**Affiliations:** 1 INEFC-Barcelona Research Group on Sport Sciences (GRCE), National Institute of Physical Education of Catalonia (INEFC), University of Barcelona (UB), 08038 Barcelona, Spain; javierespasa@roadtoperformance.com (J.E.-L.); mcarrascom@gencat.cat (M.C.-M.); 2 Physical Preparation and Load Monitoring Department, Football Club Barcelona, 08028 Barcelona, Spain; 3 Barça Innovation Hub, Football Club Barcelona, 08028 Barcelona, Spain; 4 Road to Performance Center, 15007 Coruña, Spain; 5 Department of Education, Faculty of Education Sciences, University of Almeria, 04120 Almería, Spain; carloscmrubio@gmail.com; 6 SPORT Research Group (CTS-1024), CIBIS, Research Center, University of Almería, 04120 Almería, Spain; 7 Health Research Centre, University of Almería, 04120 Almería, Spain; jol908@ual.es; 8 Department of Physical Education and Sport, Faculty of Education and Sport, University of the Basque Country, 01007 Vitoria, Spain; julio.calleja.gonzalez@gmail.com; 9 Faculty of Kinesiology, University of Zagreb, 10110 Zagreb, Croatia; 10 FPCEE Blanquerna, SAFE Research Group, Ramon Llull University, 08022 Barcelona, Spain; 11 Segle XXI Female Basketball Team, Catalan Federation of Basketball, 08950 Esplugues de Llobregat, Spain

**Keywords:** external load, basketball performance, wearable sensors, EPTS

## Abstract

Load monitoring has been identified as a valuable tool for optimizing training planning and minimizing injury risk. This study’s aim was divided into two main objectives: (1) to describe the physical demands during official competition through IMU (inertial movement unit) metrics and (2) to investigate the relationship between basketball statistics and these physical demands. Twelve female highly trained basketballers (26.5 ± 5.3 years, 180 ± 7.1 cm, and 73.6 ± 10.3 kg) were monitored during four official games. Our results indicate that games with more frequent possession changes, particularly those driven by steals and turnovers, exhibit higher physical demands. Additionally, longer game durations were associated with longer recovery time while maintaining similar active time and physical load. Players who assume prominent shooting roles face greater conditional demands, such as increased jumps and impacts, even with equal playing time. These findings suggest that IMUs provide valuable insights into high-intensity actions and patterns, indicating a direct association between physical load and player performance in professional female basketball. This study also highlights the potential for professionals to better manage workload and understand player demands using these insights, even in the absence of in-game sensor data. Our research underscores the importance of contextual analysis in sports performance studies, encouraging future investigations into game phases and their specific physical demands.

## 1. Introduction

Female basketball is characterized by high physical demands, particularly by frequent changes in direction, acceleration, deceleration, jumps, sprints, and contact, combined with aerobic and anaerobic requirements and specific individual skills [1]. Additionally, it entails complex technical–tactical skills, which impose considerable coordination demands and directly influence the physical requirements [2]. All these demanding performance aspects have spurred significant interest in monitoring the external load to optimize training regimens and mitigate injury risks [3]. Moreover, there is a growing emphasis on enhancing performance in official competition [4,5]. However, in professional female basketball, the evidential base regarding physical demands remains comparatively sparse in contrast to men’s basketball [6,7,8] and other sports [9]. This scarcity arises from limited economic resources required for acquiring necessary monitoring instruments like Electronic Performance-Tracking Systems [6]. Consequently, access to specific knowledge aimed at enhancing performance and reducing injury risks in female basketballers is currently constrained.

To the best of the authors’ knowledge, among the available scientific studies that analyze external load in female basketball, inertial measurement units (IMUs) have been used more frequently than positioning systems [6]. Although the signal processing of IMUs is still discussed by the scientific community (the sample rate setting or the individual device error) [10], manufacturers have improved this device in recent years for quantifying the physical demands through intensity (e.g., jumps or impacts) and volume metrics (e.g., player load or steps). Moreover, IMUs offer significant flexibility for its use in indoor settings compared to positioning systems, which may require an installation process [11], specifically in basketball; two recent scientific publications have used accelerometer data to gain a deeper and a more detailed understanding of the physical demands throughout the game [12,13]. This can be impractical in sports contexts in which numerous games are played away from the home venue. In contrast, IMUs do not require any installation process since the inertial sensors are embedded in a wearable device which is given to the player [11]. Furthermore, new algorithms have been validated in team sports that provide information related to the jumps [14], changes in inertia [15] and active/rest time [16]. To the best of the authors’ knowledge, these new metrics have not yet been used to describe female basketball physical demands nor have these metrics been related to game performance.

Previous research has evaluated the relationship between external load and performance during male basketball competition, but no conclusive relationships between physical demands and basketball performance have been observed [17,18,19]. However, it should be noted that this research used traditional statistics. Traditional metrics like points, rebounds, and assists have transitioned to more sophisticated measures known as advanced statistics. Over recent decades, basketball’s statistics have experienced a notable evolution, which has been driven by the increase in data availability and change in analytical methodologies [20]. For example, incorporating advanced statistics such as effective field goal percentage (eFG%), points per possession (PPP), and player usage rate (PU%) leads to a more comprehensive evaluation of player efficiency and contribution to team success [21]. These advanced statistics have not only enriched our understanding of the game but also revolutionized player performance evaluation and strategic decision-making on and off the court. While traditional statistics provide a broad overview of player and team performance, advanced statistics offer deeper and more precise assessments by considering a wider range of influencing factors. These metrics enable trainers to contextualize game events in terms of playing time, possessions, and player involvement opportunities, which may closely correlate with the physical demands experienced during gameplay [22].

Understanding the relationship between load and performance could enhance the comprehension of the physical demands in female basketball. This information would help professionals with a lack of access to any type of monitoring devices (due to economic or regulation restrictions). Therefore, exploring the relationship between load and statistics is of paramount interest, specially describing high-intensity actions, which have a major impact on the fatigue of female basketball players [5]. However, to gain a deeper understanding of physical demands and their relationship to performance, it is necessary to know contextual information about matches (e.g., home/away, final score, ranking, etc.) and individual player participation (total time, number of rotations, etc.) [23], which is often under-reported in the scientific literature.

Therefore, this study presents two main objectives. Firstly, the match physical demands of female basketball players are described using IMU metrics. This objective includes the analysis of physical demand values and the assessment of differences across matches, quarters, and playing positions. Secondly, the relationship between basketball statistics and the physical demands during the competition is investigated.

## 2. Materials and Methods

### 2.1. Participants

Twelve semi-professional and highly trained [24] female basketball players (mean age: 26.3 ± 5.3 years, mean height: 180 ± 7.1 cm, mean body mass: 73.6 ± 10.3 kg) from a 2nd Spanish League Team participated in this study (from March 2022 to May 2022). The players were categorized by coaches as follows: (1) guards; (2) forwards; and (3) centers. All participants were healthy before starting data collection. This study was approved by the Ethics Committee for Clinical Research of the Sports Administration of Catalonia (013/CEICGC/2022) and followed the ethical standards of the Committee for Responsible Human Experimentation (established by Law 14/2007, Spain) and the principles of the Declaration of Helsinki [25]. Before collecting data, all players were informed about the research procedures and agreed to participate providing written consent. The data obtained were treated with confidentially, complying with the Organic Law 15/1999 of the 13th of December on the Protection of Personal Data and the General Data Protection Regulation applicable within the European Union [26].

### 2.2. Application Procedure

A descriptive and comparative design was used to examine the relationship between game load and player performance in professional female basketball. All games were conducted following FIBA basketball rules during the 2021/22 season (from March 2022 to May 2022). Data were collected from a total of four official games, which were played at the home venue, except one away game.

**Figure 1:**
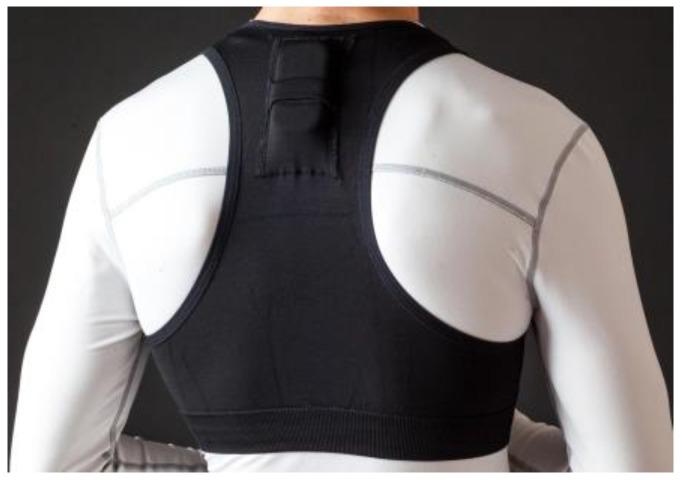
Positioning of the device in the upper back.

All players were monitored using a performance-tracking device (Wimu, Hudl^®^ SL, Lincoln, NE, USA) with a sampling frequency of 100 Hz [27]. The device (81 × 45 × 15 mm, 70 g) was placed on the upper back using an adjustable top (Figure 1). This device is equipped with a total of four accelerometers that record in different magnitude spectrums (×2: 16 G; ×1: 32 G; ×1: 400 G at 1000 Hz), three gyroscopes (×2: ±2000 degrees per second; ×1: ±4000 degrees per second at 1000 Hz), a magnetometer (±8 Gauss at 160 Hz), and one barometer (±1200 mbar at 100 Hz).

The video and IMU signals were synchronized to identify game periods and player activity on the court, excluding intervals between quarters and timeouts. Subsequently, the data collected during these identified periods were incorporated into the final analysis. The active time (AT) and recovery time (RT) metrics were calculated independently of these periods, as the latter represents the time during which a player was not active [16]. All data were downloaded from the devices and treated using the specific software of manufacturer, SPRO^TM^ (version 2.1.0, Wimu, Hudl^®^ SL, Lincoln, NE, USA) before specific statistical software was used.

#### 2.2.1. Dependent Variables

The variables reported by the sensors included in the analysis were as follows: player load (PL), high-intensity player load (Hi-PL) [28], dynamic stress load (DSL) [29], jumps, jumps’ high-intensity takeoff (Hi-Takeoff), jumps’ high-intensity landing (Hi-Landing) [14], high-intensity horizontal impacts (Hi-HI) [30], steps [31], the sum of change in inertia (COI), and the sum of high-intensity change in inertia (Hi-COI) [15]. All these variables were divided by played time. The players’ activity time was evaluated through fast Fourier-transform [16], and by calculating the difference with respect to the total time, the downtime was identified.

#### 2.2.2. Independent Variables

The statistical data were collected from the official website of the Spanish Basketball Federation (https://baloncestoenvivo.feb.es/, accessed on 10 April 2023), storing event logs and time in a customized Excel spreadsheet^®^ (Microsoft Corporation, Redmond, Washington, DC, USA).

The variables used for game performance analysis were the following: (1) total minutes; (2) points scored; (3) free throws made and attempted (1FG: M/A); (4) two-point field goals made and attempted (2FG: M/A); (5) three-point field goals made and attempted (3FG: M/A); (6) offensive rebounds (OR); (7) defensive rebounds (DR); (8) total rebounds (TR); (9) assists; (10) steals; (11) turnovers; (12) fouls committed (FC); (13) fouls received (FR); (14) efficiency rating (ER); and (15) balance. From these data, advanced statistical variables were calculated considering possessions and total possibilities in each action. The variables used were as follows: (1) effective field goal percentage (eFG%); (2) true shooting percentage (TS%); (3) assists–turnover ratio (A/TO); (4) offensive rebound percentage (ORB%); (5) defensive rebound percentage (DRB); (6) possessions; (7) points per possession (PPP); and (8) player usage percentage (PU%). The equations used to calculate the advanced stats were those proposed by the National Basketball Association [21].

Finally, player position was considered a categorical independent variable, classifying players into distinct roles based on their tactical function on the court. Additionally, game quarters and different games were treated as temporal independent variables, allowing us to examine how physical demands fluctuate over the course of the match and across different games.

### 2.3. Statistical Analysis

Descriptive (mean ± standard deviation) and normality tests (Shapiro–Wilk) were initially performed for all variables. Normality was met for PL, PL/min, Hi-PL/min, Hi-HI, Steps/min, Hi-COI, and Hi-COI/min, while the other variables did not conform to normality. Thus, in each data cluster analysis (games, quarters, and positions), normality tests were conducted to determine the data distribution for each case. Parametric and non-parametric tests were performed in each case. Differences between games and positions were assessed using one-way analysis of variance and Kruskal–Wallis in non-normal distribution cases [32]. For normal distribution variables, Levene’s test was performed to check the previous homogeneity of variances, using Fisher’s and Welch’s analysis for homogeneous and non-homogeneous variances, respectively [32], and post hoc analyses were performed to identify among which groups differences were detected, using Tukey’s test for equal variances and the Games–Howell test for different variances [32]. For the variables for which differences among groups were observed using the Kruskal–Wallis test, post hoc Dwass–Steel–Critchlow–Fligner pairwise comparison analyses were performed [32].

Bivariate and partial correlations were evaluated through Pearson’s or Spearman’s test (*r* or Rho, respectively). The strength of the correlation coefficients was interpreted as *small* (0–0.3), *moderate* (0.31–0.49), *large* (0.5–0.69), *very large* (0.7–0.89), and *near perfect* (0.9–1.0) following Cohen’s scale [33]. Partial correlations analysis was performed controlling by played time variable. Statistical significance was set at *p* < 0.05. All data were analyzed using the Jamovi project (2023), Jamovi^®^ (Version 2.3) retrieved from [34].

## 3. Results

To provide a comprehensive contextualization of the team and player data, this study details the contextual games factors (Table 1) and player participation metrics (Table 2). These metrics include the number of games played, total playing minutes, minutes played per quarter, and the number of rotations performed. Additionally, active and recovery times recorded by IMUs and the ratio between them are presented (Table 2).

Among the participants, a total of 11 players eventually took part, and not all completed the four games analyzed in this study (due to technical decisions or discomfort/injury). Furthermore, in the data collection process, some data were missed due to different reasons (improper use of the devices or failure in data collection). Of the total data anticipated prior to the commencement of the study (n = 48), a total of 38 records were incorporated into the analysis. In addition, a total of 141 records of the players’ participation in the different quarters were extracted from the competition data.

### 3.1. Physical Demands during Competition

#### 3.1.1. Analysis by Games and Quarters

The mean values and standard deviation of the absolute and relative metrics by games and quarters are shown in Table 3 and Table 4, respectively.

The PL/min was the only dependent variable which showed a significant difference among the quarters through Welch’s test (F = 3.54, *p* = 0.019). However, not significant differences were identified in post hoc analysis using Games–Howell’s test for different variances. When analyzing the differences between games, the only metric that showed significant variation was RT, specifically between games 2 and 3 (W = −3.95, *p* = 0.027) and games 3 and 4 (W = 3.70, *p* = 0.045). The remaining variables analyzed did not exhibit any significant differences between games. In the analysis by quarters, the variables RT and AT/RT showed significant differences between groups. RT demonstrated differences between Q1 and Q2 (mean difference = −5.50, *p* < 0.001), Q1 and Q3 (mean difference = −3.22, *p* < 0.001), Q1 and Q4 (mean difference = −4.48, *p* < 0.001), and Q2 and Q3 (mean difference = 2.28, *p* = 0.019). In case of the AT/RT variable, differences between Q1 and Q2 (mean difference = −3.66, *p* = 0.048) and Q1 and Q4 (mean difference = −3.97, *p* = 0.025) were identified.

#### 3.1.2. Analysis by Positions

The analysis of physical demand metrics revealed significant differences across player positions (Table 5). Regarding absolute metrics, in DSL, significant differences were found between guards and forwards (W = −3.46, *p* = 0.038). The Hi-HI showed significant differences between guards and forwards (df = 2.21, *p* = 0.008). In terms of steps per minute, significant differences were observed between guards and centers (df = 26.4, *p* = 0.002) and between forwards and centers (df = 20.8, *p* = 0.012). Steps also showed significant differences between guards and centers (W = −4.28, *p* = 0.007). Finally, significant differences in COI were observed between guards and forwards (W = −5.33, *p* < 0.001) and Hi-COI revealed significant differences between guards and forwards (df = 8.41, *p* < 0.001).

For relative metrics, PL/min showed significant differences between guards and forwards (df = 0.38, *p* = 0.015) and between guards and centers (df = 0.45, *p* = 0.013). While the Hi-PL/min indicated potential differences among groups, these were not statistically significant (df = 0.03, *p* = 0.053–0.953). Moreover, DSL/min showed significant differences between guards and centers (W = −3.54, *p* = 0.033). Hi-Takeoff/min revealed significant differences between guards and forwards (W = −3.58, *p* = 0.030), while Hi-HI/min exhibited significant differences between guards and forwards (W = −4.12, *p* = 0.010). Lastly, COI/min showed significant differences between guards and forwards (W = −6.48, *p* < 0.001), as well as between guards and centers (W = −4.04, *p* = 0.012). These results underscore the positional variations in physical demands within the context of female basketball.

### 3.2. Correlation between Physical Demands and Game Performance

The results of the bivariate and partial correlation analyses between physical demands and game performance are presented in Table 6.

The bivariate correlations revealed that the statistical variables most correlated with the IMU metrics were steals and turnovers. Both variables exhibited eight significant correlations of moderate to large magnitude (steals: 0.37–0.58; turnovers: 0.39–0.65). When conducting partial correlation analyses controlling for the variable “played time”, the variables of steals (four moderate correlations: 0.37–0.48) and turnovers (six correlations of moderate to large magnitude: 0.34–0.59) continued to show the most significant correlations with the physical demand metrics, although with a reduced number and magnitude. Points and jumps showed a significant correlation too (0.34).

The bivariate correlation analysis among points, shot attempts and physical demands showed significant moderate-to-large correlations. In case of points and external load, correlations were identified with jumps (0.56), Hi-Landing (0.42), and PL (0.34). Secondly, 1FG correlated to Hi-COI (0.56), while 2FG and 3FG showed a greater number of relationships. The 2FG correlated to jumps (0.52), Hi-Landing (0.50), Hi-HI (0.44) and COI (0.35), and 3FG correlated with steps (0.40), COI (0.41) and PL (0.44). All these relationships were not observed in the partial correlations (1FG and Hi-COI: 0.57; 2FG and Hi-Landing: 0.46 and Jumps: 0.44). Regarding the advanced statistical metrics, few significant relationships were observed, all of which were of a moderate level (0.33–0.45). Notably, the number of possessions was correlated with Hi-PL (0.35), DSL (0.35), and steps (0.45). Points per possession were correlated with Hi-Landings (0.33) and Hi-COI (0.33), while Player Usage (PU) was correlated with Hi-Landings (0.44) and Hi-HI (0.40). Additionally, the percentage of offensive rebounds and the number of steps showed the only significant negative moderate correlation identified through the bivariate correlation analysis (−0.35).

The amount and magnitude of these inverse correlations increased in the partial correlation analysis. The steps variable demonstrated the highest number of significant inverse relationships, with moderate-to-large magnitudes, with statistical metrics (points: −0.54; PPP: −0.52; ER: −0.52; TS: −0.47; OppF: −0.43; eFG: −0.38).

Additionally, the correlation between total playing time and the active time reported by the device was evaluated, and moderate and large significant correlations were found in case of game (Rho = 0.42 *p* = 0.008) and quarter (Rho = 0.60, *p* < 0.001) analysis, respectively.

## 4. Discussion

The present study presented two main objectives: (1) to describe the physical demands of female basketballers during matches using IMU metrics, and (2) to investigate how basketball performance statistics relate to these physical demands during official competition. To the best of the researchers’ knowledge, this is the first study that provides a physical demands’ description of female basketball competition, adding a contextual description of the games. This study included information such as game outcomes, team standings and number of players (over total available roster). Furthermore, individual participation is described by game and quarter, incorporating a novel indicator: the number of rotations, or instance in which a player enters the court from the bench. On the other hand, novel IMU variables not previously used in this population assessment were analyzed (such as DSL, COI, or AT).

The findings suggest that IMU metrics provide relevant information about high-intensity actions in female basketball and reveal consistent patterns indicative of a direct association between physical load and player performance in professional female basketball. In this study, differences among games, quarters and positions were identified. Also, correlation analysis showed moderate and large correlations between physical load and game performance.

The main findings of this study indicate that games with more frequent possession changes, especially those driven by higher numbers of steals and turnovers, presented greater physical demands. Longer game durations were associated with a longer recovery time, yet similar active time and physical load. Players who take on a prominent shooting role experience higher conditional demands, such as increased jumps, falls, and impacts, even with equal playing time. These insights could help professionals understand player demands and manage workload effectively, even without access to sensors, and provide valuable information on the frequency of specific actions like impacts and jumps in female basketballers.

### 4.1. Physical Demands during Competition

Each basketball game presents a unique scenario that the staff and players must prepare for in a short period of time. The differences in tactical preparation, considering the opponent’s strategy, cause the demands of each game to vary. Despite this, when differences were evaluated among the games, the RT variable showed statistically significant differences between matches. This metric allows for the estimation of the players’ resting time through the intensity of fast Fourier-transform of the accelerometer [16] (Table 1). This finding related to game differences may highlight two key aspects: (1) differences in game duration are mainly caused by more resting time, and (2) physical demands appear to be consistent regardless of the opponent. According to the first point, it is noteworthy that the longest games were those with a smaller score difference (Game 2 and Game 4). Possible explanations about these findings include the occurrence of more time stops (e.g., fouls, time-outs) during moments of minimal score difference, allowing players to recover during these breaks [35]. This phenomenon appears to be replicated when studying the differences among quarters, showing that Q1 is statistically different from the rest (mean difference range = −5.50 to −2.28) with lower average values (9.4 ± 2.2 compared to 14.9 ± 3.8, 12.6 ± 2.4, and 13.9 ± 3.5 min). This could be due to a lower level of fatigue among players, who may try to elevate the pace of the game in the first minutes and even attempt to participate more in offensive and defensive situations. This initial hypothesis, which agrees with values already reported in the literature [36], should be further investigated in the future, as the authors have not found studies in basketball that have analyzed these novel variables, which are potentially useful in the study of physical demand and the management of fatigue and recovery.

Only the variable PL/min showed significant differences among quarters when performing one-way ANOVA analysis; however, no differences were identified among the groups in the post hoc tests. These results differ from those obtained by Portes et al. (2022), who studied physical demands using variables common to this research and employing the same devices in young female basketballers [37]. In their results, PL and PL/min showed statistically significant differences between games and quarters for guard, forward, and center positions. Despite this, it should be noted that in the tables reported in Portes et al. (2022), the standard deviation values for these variables exceeded their mean values (e.g., guards: 7.1 ± 7.4; see Table 2). This fact could indicate a wide dispersion of the data mainly due to methodological differences in managing active and rest times, given that similar differences were observed with other variables analyzed in both studies, such as jumps and jumps/min. Garcia et al. (2020) and Pérez-Chao et al. also describe differences in the variable PL among quarters when studying young male basketball players [36,38]. In the first case, the authors reported only per-minute data (a common highlighted result), while in the second case, physical demand values were evaluated in different time windows (30 s, 45 s, 1 min, 2 min, and 5 min). Although differences in the studied samples could be a critical factor in understanding the discrepancies among the results, it is essential to clarify the signal treatment with precision. This involves providing detailed information on which moments of the game were included in the analysis and which were excluded. Additionally, it is crucial to report comprehensive data on individual player participation (e.g., Table 2).

When analyzing physical demands by player positions, the number of differences among groups increases compared to the analysis by quarters. Specifically, guards exhibit more differences compared to forwards and centers than those between forwards and centers, with the latter groups differing only in the variable steps/min. Guards stand out as the group with the highest average values in most physical demand variables. These values were significantly different in 11 of 23 metrics studied: PL/min, DSL, DSL/min, Hi-Takeoff/min, Hi-HI, Hi-HI/min, steps, steps/min, COI, COI/min, and Hi-COI.

Among all the variables used to describe physical demands, two distinct groups can be identified: those related to volume and those related to high-intensity actions. For volume-related variables, such as PL/min and steps/min, previous studies have shown correlations with the total distance covered [23,39]. In this study, players with the highest average relative values per minute for these movement-related variables were guards (PL/min: 2.5, steps/min = 67.3) and forwards (steps/min: 59.5). These findings are consistent with the existing scientific evidence [40,41,42]. Thus, other variables as DSL/min and COI/min showed different values among positions, pointing to guards receiving more impacts and developing more changes in direction during the game. These results might be attributed to the specific game dynamics. Guards are responsible for ball handling and moving across the entire court [40], while forwards engage in more movements during static half-court situations to create opportunities for themselves or their teammates. Defensively, guards might also cover more ground due to their role in pursuing opponents across the full court. In contrast, centers often move in straight lines to either end of the court to attack or defend, spending more time in static positions or in smaller areas while executing tactical plays [36]. These findings indicate that outside players have a greater amount of movement than inside players [36]. These contextual analyses based on game phases are already conducted in other sports and it could be beneficial to apply these methods to female basketball [43].

On the other hand, intensity-related variables such as the number of jumps (and their phases: Hi-Takeoff and Hi-Landing) and Hi-COI, both absolute and per minute, were also examined. Although the variable for total jumps did not show significant differences, guards performed the most jumps during the games, while forwards performed the least (guards = 13.4, forwards = 7.7, centers = 11.5). These values differ from those previously reported by IMU in young female players when studying positional differences [37,44] but are similar in total number to those reported through video analysis in professional and elite-level players [45,46]. The observed discrepancies may be due to methodological differences and the new algorithm used during this study that provides more reliable [14] results and similar to those reported with video analysis [47]. In addition, this study examines the phases of jumping identified by inertial sensors in female basketball players separately. The analysis revealed that guards exhibited a higher number of high-intensity takeoffs per minute (Hi-Takeoff/min = 0.2) compared to other positions. In case of Hi-COI, this metric also showed higher averages among guards and centers, although data dispersion was much higher for centers (9.61 ± 6.6 vs. 13.51 ± 24.7). This likely led to significant differences being observed only between guards and forwards. To the authors’ knowledge, this is the first time COI data are published about female basketballers during competition, preventing direct comparisons with the existing literature. However, the observed differences could be attributed to specific roles and actions during the game. The assigned tasks and lateral dominance may significantly influence the total and the direction of COI, a phenomenon that has been observed in soccer players [47,48]. For guards, dribbling during offensive play and the need to change direction in defensive situations to maneuver around or stop opponents likely contribute to these demands. In the case of centers, both the average values and the standard deviation could be explained by different play styles into the same position (e.g., fighters: players who prefer playing with their backs to the basket and maintaining contact with their defenders; or shooters: players who avoid contact by shooting from a distance or engaging in 1v1 situations facing the basket). This idea is supported by the non-significant differences observed in the games studied. For instance, the second game, the most closely contested, showed the highest physical demands in high-intensity changes in direction for the entire team (14.6 ± 24.2). Future research is warranted to further explore the relationship between game outcomes and high-intensity actions in order to enhance the understanding and interpretation of these findings.

### 4.2. Physical Demands and Performance during Competition

Few studies in the scientific literature examine the relationship between physical demands and performance during basketball competition, both for female players [49,50] and male players [17,18]. These studies generally report limited correlations between game performance and physical demands, consistently highlighting the variable PL in line with our results. Among the two studies that investigated the relationship between physical demands and performance in women’s basketball, only one reported a significant relationship. Gasperi et al. (2023) used PL/min as the individual external load metric during competition analysis [50]. They found no significant relationships between PL/min and variables such as eFG% or the total rebounds. Conversely, Brown et al. (2023), reported significant correlations between PL and PL/min with game performance metrics (points: 0.61, field goals: 0.41, and 1FG: 0.38), which differ from the findings of this study (points: 0.34, 1FG: 0.06) [18]. These discrepancies may be attributed to methodological differences, such as the inclusion of timeouts in the data analysis. Additionally, the correlations between jumps and COI with basketball game performance can only be compared to one available publication, which did not report significant correlations (regardless of the threshold or direction of these actions) [18].

To the authors’ knowledge, this publication includes the highest number of physical demand variables and game statistics (and it is the only one to analyze advanced statistical parameters) available in the literature related to female basketballers. In the analyses of the relationships among them, different magnitudes of correlation were observed, with steals and turnovers emerging as the variables most frequently correlated with physical demands (bivariate and partial correlations). This could be easily explained: when either of these situations occurs during a game, it counts as a change in possession, resulting in a shift in gameplay where the team that was attacking now defends, and vice versa (correlation between COI and S and TO: 0.52 and 0.46, respectively). This increase in the number of possessions during the game could explain other identified relationships, as the players should require covering more distance (relationship between total steps and number of possessions: 0.45) and perform more high-intensity actions (relationship between steals, turnovers, and number of possessions with Hi-PL and jumps variables). Additionally, the number of 2FG was correlated with jumps’ parameters, such as total jumps (bivariate correlation: 0.52, and partial correlation: 0.44) and Hi-Landing (bivariate correlation: 0.50, and partial correlation: 0.46). This could suggest that many of the shots are executed while jumping, with layups potentially involving high-impact landings [51]. However, future studies should further investigate these relationships to elucidate their underlying mechanisms.

Although these are the first data presented in the study of female basketball, a very interesting significant inverse relationship was detected between the variable steps and PPP (−0.52), ER (−0.52), TS (−0.47), and eFG (−0.38). This phenomenon may indicate that the steps variable, previously identified as a measure of the amount of movement during the game, could be related to game efficiency. Higher scoring accuracy might be associated with lower physical demands. This phenomenon was observed in partial correlation but not in bivariate correlation analysis. This could be due to participation game time as a key factor during the study of physical demands.

Despite finding significant correlations, they are of moderate to large magnitude, indicating the need for further research to clarify how physical demands relate to game performance. Future studies should evaluate specific phases of the game, such as offense and defense independently, and discuss the necessity of including or excluding activity during stoppages (e.g., inbound plays). Variables of great interest, like active time and rest time, should be included in future research.

Additionally, the relationship between the reported playing time statistics and the active time recorded by the devices was analyzed. Although this relationship was significant, its value was moderate and large in the overall game analysis (Rho = 0.42, *p* = 0.008) and per quarter (Rho = 0.60, *p* < 0.001), respectively. This finding suggests that considering only the reported playing time may not be sufficient. Despite excluding pre-game warm-ups, quarter breaks, and timeouts from this analysis, players may still accumulate load while the game clock is stopped, such as during inbound and sideline plays. Monitoring these game moments, which might sometimes be decisive for the outcome, could be highly beneficial. Therefore, players wearing IMUs that calculate the total active time might be of interest for more precise load monitoring.

### 4.3. Limitations, Future Directions and Practical Applications

Although the objective of this study—analyzing physical demands during official competition in professional female basketball players—was achieved, several limitations must be acknowledged. The primary one is the sample size. Thus, these results should be interpreted with caution when considering their generalizability. While the study population is relatively inaccessible, and the games analyzed were against teams with varying rankings, the total number of games represents only 13% of the total played games into the season (in the end of competitive period). Additionally, all the games resulted in a loss, and three were played at home. Although this study reported contextual values for the teams, differences in scores, and final statistics, it did not account for the statistics of the opposing teams. Addressing this gap, along with monitoring the physical demands of the rival team’s players, could provide a more comprehensive understanding of the physical demands and their relationship to individual and team performance, as well as score differences.

Future research should include a longitudinal monitoring approach to better understand the physical demands in professional female basketball competition. Additionally, expanding the sample size and considering the performance metrics of opposing teams would enhance the robustness and applicability of the findings.

Despite the limitations and the need for further research, these findings offer valuable insights for optimizing training programs to address position-specific physical demands. By identifying players who may accumulate higher physical loads during competition—whether due to their tactical role, increased playing time, or games with more frequent possession changes due to steals or turnovers—coaches can better manage player efforts during games. This approach is especially critical for players with a high volume of shots, as it helps to avoid excessive fatigue and reduce the risk of injury. Additionally, these insights support the development of individualized recovery strategies post competition. It is important to highlight that these recommendations could be particularly beneficial for environments without access to advanced load monitoring technology, enabling coaches to more effectively manage player workload and recovery by understanding the relationship between physical load and game performance.

## 5. Conclusions

This study highlights the importance of considering positional and game periods differences, adding game context, when analyzing physical demands in female basketball official competition.

In relation to the first objective, this study provides a comprehensive description of the physical demands during official competition through IMU (inertial movement unit) metrics. The observed relationships between physical demands and performance suggest that games with greater changes in possession, particularly those caused by a higher number of steals and turnovers, exhibit higher physical demands. This information provides professionals without access to this technology with useful indicators for better load management, whether by adjusting substitutions during the match (to replace the most fatigued players) or by adapting the training load the following day (either decreasing or increasing it as needed). Addressing the second objective, the findings indicate that a longer duration of the games was associated with more recovery time, while similar active time and physical load were observed. Furthermore, given equal playing time, players who take on a more prominent role in shooting may experience greater conditional demands (such as a higher number of jumps, falls, or impacts). Additionally, these insights provide valuable information on the frequency of specific actions in female basketball (impacts, jumps, etc.).

Despite the limitations, these findings help customize training programs to position-specific demands, identify players at risk of high physical loads, and optimize and individualize recovery strategies. This approach is crucial for managing load process, especially in teams with no possibility of accessing this technology, allowing the staff to prevent fatigue and injuries during the season. Future research should continue to explore these relationships by incorporating advanced statistical methods and a broader range of performance metrics and include contextual analysis as attack/defense description or study the demands during a game’s stopped-ball moments.

## Figures and Tables

**Table 1:** Contextual data of games and team’s stats.

Variable	Game 1	Game 2	Game 3	Game 4	All Games
**Game contextual data**					
Home/Away	Home	Home	Home	Away	-
Round (over 30 games)	24	22	26	27	-
Ranking (over 16 teams)	13 vs. 8	13 vs. 3	13 vs. 14	1 vs. 13	-
Score (points)	38/55 (−17)	57/59 (−2)	50/64 (−14)	68/61 (−7)	213/239 (sum)
Win/Lose	Lose	Lose	Lose	Lose	0/4 (sum)
PP/TP (No.)	11/12	8/12	9/12	10/12	38/48 (sum)
TT (min)	95.3	106.9	90.9	100.9	98.5 (mean)
**Team’s traditional and advanced statistics (sum and mean values)**					
1FG (M/A; %)	9/10; 0.90	11/18; 0.61	7/13; 0.54	14/22; 0.64	41/63; 0.65
2FG (M/A; %)	13/40; 0.36	17/34; 0.50	11/31; 0.35	13/36; 0.36	54/141; 0.38
3FG (M/A; %)	1/20; 0.05	2/20; 0.10	3/20; 0.15	7/18; 0.39	13/78; 0.17
TR: OR/DR (No.)	34: 11/23	32: 10/22	34: 15/19	25: 6/19	125: 42/83
Sum of Assists. (No.)	6	12	9	10	37
Sum of Steals. (No.)	12	6	5	16	39
Sum of TO (No.)	18	14	9	16	57
Sum of FC (No.)	17	14	10	17	58
Sum of FR (No.)	15	18	14	21	38
Sum of ER (AU)	24	49	39	60	172
Mean of +/− (AU)	−7.73	−2.13	−3.89	−3.50	−4.31
Mean of Possessions (No.)	37.7	34.3	37.6	38.4	37.2
Mean of PPP (AU)	0.16	0.30	0.17	0.18	0.20
Mean of eFG (%)	0.21	0.28	0.28	0.41	0.30
Mean of ORB (%)	0.06	0.07	0.08	0.06	0.07
Mean of DRB (%)	0.14	0.18	0.16	0.12	0.15
Mean of TS (%)	0.26	0.30	0.32	0.46	0.34
Mean of A/TO	0.31	0.78	0.93	0.55	0.64
Mean of PU (%)	0.19	0.22	0.20	0.18	0.20

+/−: player balance; A/TO: assists–turnover ratio; DR: defensive rebounds; DRB: defensive rebound percentage; eFG: efficiency field goal percentage; ER: efficiency rating; FC: fouls committed; FG: field goals (made/attempted); FR: fouls received; OF: offensive rebounds; ORB: offensive rebound percentage; PP: number of players who participated; PPP: points per possession; TP: total of players available for playing; TR: total rebounds; USG: usage percentage; AU: arbitrary units.

**Table 2:** Individual data of player’s participation by game and quarters.

Player (Position)	Game	Initial or Bench	PT (min)	No. of Rotations	No. of Rotations per Quarter; PT per Quarter (min)			
					Q1	Q2	Q3	Q4
P1 (G)	1	Bench	14.5	7	3.9; 1	2.9; 2	5.2; 2	2.5; 2
P1 (G)	2	Initial	17.1	3	9.4; 1	0.8; 1	6.9; 1	-
P1 (G)	3	Initial	20.8	6	4.9; 1	7.8; 1	2.9; 2	5.1; 2
P1 (G)	4	Bench	17.4	4	5.1; 1	6.0; 1	1.4; 1	4.9; 1
P2 (G)	1	Initial	10.7	2	6.1; 1	4.6; 1	-	-
P2 (G)	2	Bench	11.3	4	0.7; 1	3.2; 1	3.1; 1	4.3; 1
P2 (G)	3	Bench	17.5	4	5.1; 1	2.2; 1	7.1; 1	3.1; 1
P2 (G)	4	Bench	23.0	7	4.9; 1	4.3; 2	9.1; 2	4.7; 2
P3 (G)	1	Bench	4.6	2	-	-	3.1; 1	1.5; 1
P3 (G)	3	Bench	14.0	3	2.9; 1	8.0; 1	-	3.1; 1
P4 (G)	1	Bench	28.0	5	4.8; 1	5.4; 2	9.3; 1	8.5; 1
P4 (G)	2	Bench	24.8	7	3.7; 1	7.5; 2	4.9; 2	8.7; 2
P4 (G)	3	Bench	18.9	7	5.1; 1	4.9; 3	1.9; 2	6.9; 1
P4 (G)	4	Bench	16.6	6	3.9; 1	0.7; 1	6.2; 2	5.8; 2
P5 (G)	1	Initial	24.6	4	5.2; 1	7.1; 1	4.7; 1	7.5; 1
P5 (G)	2	Initial	31.1	6	6.8; 1	7.8; 1	8.3; 2	8.2; 2
P5 (G)	4	Initial	29.7	4	6.1; 1	9.1; 1	6.3; 1	8.2; 1
P6 (F)	1	Bench	7.3	2	3.9; 1	3.4; 1	-	-
P6 (F)	2	Initial	9.7	2	5.1; 1	-	4.6; 1	-
P6 (F)	3	Bench	4.2	1	-	4.2; 1	-	-
P6 (F)	4	Initial	9.2	2	5.1; 1	4.1; 2	-	-
P7 (F)	1	Initial	22.7	4	7.4; 1	3.5; 1	5.9; 1	6.0; 1
P7 (F)	3	Initial	18.7	4	4.9; 1	5.8; 1	1.2; 1	6.9; 1
P7 (F)	4	Initial	19.2	6	5.5; 1	3.0; 2	3.7; 2	7.0; 1
P8 (F)	1	Bench	18.7	5	2.6; 1	0.1; 1	4.1; 1	5.4; 1
P8 (F)	3	Bench	21.3	5	5.1; 1	4.2; 2	8.9; 1	3.1; 1
P8 (F)	4	Bench	21.8	6	4.6; 1	7.9; 3	6.3; 1	3.0; 1
P9 (C)	1	Initial	18.7	4	5.2; 1	4.7; 1	4.7; 1	4.0; 1
P9 (C)	2	Bench	21.7	4	2.0; 1	4.9; 1	4.9; 1	10; 1
P9 (C)	3	Initial	18.3	5	5.7; 1	7.1; 1	2.9; 1	2.6; 1
P9 (C)	4	Initial	18.9	5	6.1; 1	3.5; 1	4.4; 1	5.0; 2
P10 (C)	1	Bench	19.9	5	4.8; 1	5.3; 2	5.3; 1	4.6; 1
P10 (C)	2	Initial	18.3	4	8.0; 1	5.2; 1	5.2; 1	0.1; 1
P10 (C)	3	Bench	18.6	4	4.3; 1	2.9; 1	7.1; 1	4.3; 1
P10 (C)	4	Bench	21.5	6	3.9; 1	4.2; 2	8.4; 2	5.0; 1
P11 (F)	1	Initial	30.4	5	6.1; 1	6.7; 1	7.7; 2	10; 1
P11 (F)	2	Initial	25.2	4	6.3; 1	8.5; 1	6.9; 1	3.5; 1
P11 (F)	4	Bench	22.9	7	4.9; 1	7.2; 2	4.4; 2	6.5; 2

Q: quarter; PT: played time; AT: active time; RT: recovery time; C: center; F: forward; G: guard; P: player; PF: power forward; Q: quarter; SF: small forward. Rotations: times substituted in from the bench.

**Table 3:** Descriptive and difference between absolute and relative team’s physical demands by game.

Variables	Game 1 (Mean ± SD)	Game 2 (Mean ± SD)	Game 3 (Mean ± SD)	Game 4 (Mean ± SD)	All Games (Mean ± SD)
PL (AU)	39.4 ± 19.3	47.5 ± 18.4	34.9 ± 11.5	43.7 ± 15.1	41.2 ± 16.4
PL/min (AU)	2.2 ± 0.3	2.4 ± 0.4	2.1 ± 0.4	2.2 ± 0.5	2.2 ± 0.4
Hi-PL (AU) *	2.7 ± 1.8	3.8 ± 2.0	2.4 ± 1.1	2.8 ± 1.6	2.87 ± 1.7
Hi-PL/min (AU)	2.9 ± 0.3	2.4 ± 0.4	2.1 ± 0.4	2.2 ± 0.5	0.1 ± 0.0
DSL (AU) *	78.5 ± 55.2	121 ± 78.4	76.1 ± 42.1	99.3 ± 71.8	92.4 ± 62.7
DSL/min (AU) *	2.7 ± 2.5	3.7 ± 1.7	2.8 ± 1.2	2.6 ± 1.7	2.9 ± 1.8
Jumps (No.)	11.3 ± 8.0	13.3 ± 8.0	8.6 ± 5.9	11.2 ± 9.4	11.0 ± 7.8
Jumps/min (No.)	0.2 ± 0.1	0.2 ± 0.1	0.2 ± 0.1	0.2 ± 0.1	0.2 ± 0.1
Hi-Takeoff (No.) *	4.1 ± 3.3	5.5 ± 4.5	4.9 ± 4.8	5.6 ± 5.4	5.0 ± 4.4
Hi-Takeoff/min (No.) *	0.2 ± 0.1	0.2 ± 0.2	0.2 ± 0.1	0.2 ± 0.2	0.2 ± 0.1
Hi-Landing (No.) *	5.9 ± 4.5	7.4 ± 5.1	4.1 ± 3.8	6.0 ± 5.8	5.8 ± 4.8
Hi-Landing/min (No.) *	0.2 ± 0.1	0.3 ± 0.2	0.1 ± 0.1	0.2 ± 0.1	0.2 ± 0.1
Hi-HI (No.)	3.3 ± 2.5	4.6 ± 2.3	2.4 ± 2.1	2.1 ± 2.3	3.1 ± 2.4
Hi-HI/min (No.) *	0.1 ± 0.0	0.1 ± 0.1	0.1 ± 0.1	0.1 ± 0.0	0.1 ± 0.1
Steps (No.) *	1867 ± 984	1978 ± 1091	1586 ± 565	2115 ± 752	1889 ± 855
Steps/min (No.)	57.0 ± 12.8	61.9 ± 23.7	57.1 ± 12.5	57.1 ± 12.9	58.1 ± 15.1
COI (No.) *	141 ± 71.8	152 ± 62.4	131 ± 66.1	139 ± 76.1	140 ± 67.3
COI/min (No.) *	4.5 ± 1.7	4.7 ± 1.5	4.8 ± 2.3	3.6 ± 1.5	4.4 ± 1.8
Hi-COI (No.)	5.7 ± 4.7	14.6 ± 24.2	7.2 ± 9.2	5.9 ± 6.1	8 ± 12
Hi-COI/min (No.)	0.2 ± 0.1	0.4 ± 0.6	0.3 ± 0.4	0.2 ± 0.1	0.2 ± 0.3
AT (min) *	32.4 ± 16.0	29.1 ± 10.0	44.4 ± 48.1	28.2 ± 7.7	33.5 ± 25.4
RT (min) *^,†^	64.1 ± 16.0	77.7 ± 10.0	62.5 ± 5.6	72.5 ± 7.7	68.8 ± 12.1
AT/RT *	0.7 ± 0.9	0.4 ± 0.2	0.4 ± 0.1	0.4 ± 0.1	0.5 ± 0.5

SD: standard deviation; * non-normal distribution (*p* < 0.05); ^†^: significant difference between games; AT: active time; COI: changes in inertia; DSL: dynamic stress load; Hi-COI: high-intensity changes in inertia; Hi-HI: high-intensity horizontal impacts; Hi-Landing: jumps’ high-intensity landing; Hi-PL: high-intensity player load; Hi-Takeoff: jumps’ high-intensity takeoff; PL: player load; RT: recovery time; AU: arbitrary units; min: minutes.

**Table 4:** Descriptive and difference between absolute and relative team’s physical demands by quarter.

Variables	Q1 (Mean ± SD)	Q2 (Mean ± SD)	Q3 (Mean ± SD)	Q4 (Mean ± SD)
PL (AU) *	11.0 ± 3.8	12.1 ± 5.9	11.7 ± 5.6	12.8 ± 6.5
PL/min (AU) ^†^	1.8 ± 0.2	1.1 ± 0.3	1.2 ± 0.3	1.2 ± 0.3
Hi-PL (AU) *	0.8 ± 0.4	0.8 ± 0.6	0.8 ± 0.5	0.9 ± 0.6
Hi-PL/min (AU) *	0.1 ± 0.0	0.1 ± 0.1	0.1 ± 0.1	0.1 ± 0.1
DSL (AU) *	24.8 ± 16.4	28.3 ± 21.1	24.4 ± 18.0	29.1 ± 22.7
DSL/min (AU) *	3.1 ± 1.8	2.8 ± 1.6	2.5 ± 1.5	2.7 ± 1.6
Jumps (No.) *	2.6 ± 2.4	3.0 ± 2.5	3.4 ± 2.9	3.6 ± 3.3
Jumps/min (No.) *	0.3 ± 0.2	0.3 ± 0.3	0.4 ± 0.4	0.4 ± 0.3
Hi-Takeoff (No.) *	1.8 ± 1.9	1.3 ± 1.5	1.5 ± 1.8	1.2 ± 1.5
Hi-Takeoff/min (No.) *	0.2 ± 0.2	0.2 ± 0.3	0.1 ± 0.1	0.1 ± 0.2
Hi-Landing (No.) *	1.6 ± 1.7	1.6 ± 2.1	1.9 ± 1.7	1.4 ± 1.2
Hi-Landing/min (No.) *	0.2 ± 0.2	0.2 ± 0.2	0.2 ± 0.2	0.1 ± 0.1
Hi-HI (No.) *	0.8 ± 1.1	1.0 ± 1.1	0.6 ± 1.0	1.3 ± 1.5
Hi-HI/min (No.) *	0.1 ± 0.2	0.1 ± 0.1	0.1 ± 0.1	0.1 ± 0.1
Steps (No.) *	522.8 ± 191.3	542.4 ± 598.9	538.8 ± 312.8	576.3 ± 321.8
Steps/min (No.) *	65.6 ± 16.2	54.0 ± 15.5	55.6 ± 18.2	56.0 ± 19.7
COI (No.) *	36.2 ± 19.9	41.0 ± 23.0	39.0 ± 21.2	41.2 ± 22.5
COI/min (No.) *	4.6 ± 2.2	4.9 ± 4.8	4.3 ± 1.8	4.1 ± 2.2
Hi-COI (No.) *	1.8 ± 2.2	2.5 ± 4.2	2.4 ± 3.7	2.3 ± 5.5
Hi-COI/min (No.) *	0.3 ± 0.4	0.3 ± 0.4	0.3 ± 0.4	0.2 ± 0.3
AT (min) *	6.3 ± 2.0	6.9 ± 2.7	6.9 ± 2.4	6.6 ± 2.7
RT (min) ^†^	9.4 ± 2.2 ^1,2,3^	14.9 ± 3.8 ^4^	12.6 ± 2.4	13.9 ± 3.5
AT/RT *^,†^	0.8 ± 0.5 ^1,3^	0.6 ± 0.4	0.6 ± 0.3	0.5 ± 0.3

SD: standard deviation; * non-normal distribution (*p* < 0.05); ^†^: significant difference among quarters; ^1^: significant difference between first and second quarter; ^2^: significant difference between first and third quarter; ^3^: significant difference between first and fourth quarter; ^4^: significant difference between second and third quarter; AT: active time; COI: changes in inertia; DSL: dynamic stress load; Hi-COI: high-intensity changes in inertia; Hi-HI: high-intensity horizontal impacts; Hi-Landing: jumps’ high-intensity landing; Hi-PL: high-intensity player load; Hi-Takeoff: jumps’ high-intensity takeoff; PL: player load; RT: recovery time; AU: arbitrary units; min: minutes.

**Table 5:** Descriptive and difference between absolute and relative physical demands by position.

Variables	Guards Mean ± SD	Forwards Mean ± SD	Centers Mean ± SD
PL (AU)	41.9 ± 15.2	41.7 ± 21.2	38.6 ± 5.4
PL/min (AU) ^†^	2.5 ± 0.3 ^1,2^	2.1 ± 0.4	2.0 ± 0.2
Hi-PL (AU) *	3.1 ± 1.2	3.0 ± 2.3	2.3 ± 0.9
Hi-PL/min (AU) ^†^	0.1 ± 0.0	0.1 ± 0.0	0.1 ± 0.0
DSL (AU) *^,†^	103.5 ± 42.3 ^1^	96.1 ± 83.6	65.5 ± 37.2
DSL/min (AU) *^,†^	3.8 ± 2.1 ^2^	2.6 ± 1.6	2.0 ± 1.2
Jumps (No.)	13.4 ± 10.0	7.7 ± 3.6	11.5 ± 5.7
Jumps/min (No.)	0.2 ± 0.2	0.1 ± 0.1	0.1 ± 0.1
Hi-Takeoff (No.) *	6.7 ± 5.5	3.9 ± 3.3	4.1 ± 3.6
Hi-Takeoff/min (No.) *^,†^	0.2 ± 0.1 ^1^	0.1 ± 0.1	0.1 ± 0.1
Hi-Landing (No.) *	5.0 ± 3.0	6.4 ± 6.3	6.0 ± 4.1
Hi-Landing/min (No.) *	0.2 ± 0.1	0.2 ± 0.2	0.2 ± 0.1
Hi-HI (No.) ^†^	3.2 ± 1.9 ^1^	2.6 ± 2.5	3.8 ± 3.0
Hi-HI/min (No.) *^,†^	0.1 ± 0.1 ^1^	0.1 ± 0.1	0.1 ± 0.1
Steps (No.) *^,†^	2023.1 ± 719.6 ^2^	2067.7 ± 1021.6	1298.6 ± 399.4
Steps/min (No.) ^†^	67.3 ± 5.2 ^2^	59.5 ± 13.4 ^3^	39.2 ± 13.7
COI (No.) *^,†^	172.6 ± 51.7 ^1^	112.9 ± 79.9	138.4 ± 38.7
COI/min (No.) *^,†^	5.9 ± 1.3 ^1,2^	3.1 ± 1.3	4.0 ± 1.1
Hi-COI (No.) ^†^	9.6 ± 6.6 ^1^	3.8 ± 5.0	13.5 ± 24.7
Hi-COI/min (No.)	0.4 ± 0.3	0.1 ± 0.1	0.4 ± 0.6
AT (min) *	32.7 ± 13.0	27.1 ± 7.7	45.4 ± 51.7
RT (min) *	65.7 ± 14.2	70.7 ± 10.8	72.4 ± 8.6
AT/RT *	0.6 ± 0.7	0.4 ± 0.2	0.4 ± 0.2

SD: standard deviation; * non-normal distribution (*p* < 0.05); ^†^: significant difference among groups; ^1^: significant difference between guards and forwards; ^2^: significant difference between guards and centers; ^3^: significant difference between forwards and centers; AT: active time; COI: changes in inertia; DSL: dynamic stress load; Hi-COI: high-intensity changes in inertia; Hi-HI: high-intensity horizontal impacts; Hi-Landing: jumps’ high-intensity landing; Hi-PL: high-intensity player load; Hi-Takeoff: jumps’ high-intensity takeoff; PL: player load; RT: recovery time; AU: arbitrary units; min: minutes.

**Table 6:** Bivariate and partial correlation levels between absolute physical demands and game performance.

Variables	PL ^a^	Hi-PL ^β^	DSL ^β^	Jumps ^β^	Hi-Takeoff ^β^	Hi-Landing ^β^	Hi-HI ^a^	Steps ^β^	COI ^β^	Hi-COI ^a^
Bivariate correlations										
Points	0.34 *	0.26	0.09	0.56 ***	0.25	0.42 **	0.29	0.06	0.25	0.24
1FG (A)	0.06	0.03	−0.14	−0.11	−0.11	−0.20	0.21	−0.13	0.05	0.56 **
2FG (A)	0.33	0.26	0.20	0.52 **	0.33	0.50 **	0.44 *	0.00	0.35 *	0.11
3FG (A)	0.44 *	0.29	0.25	0.31	0.20	0.33	0.30	0.40 *	0.41 *	0.15
OR	−0.06	−0.07	−0.18	−0.01	0.02	−0.13	0.01	−0.18	−0.04	−0.01
DR	0.23	0.32	0.15	0.36 *	0.21	0.22	0.37	0.13	0.14	0.24
TR	0.15	0.18	0.02	0.24	0.12	0.05	0.29	−0.01	0.08	0.18
Assists	0.32	0.33 *	0.17	0.06	0.10	0.17	0.15	0.31	0.37 *	0.05
Steals	0.41 *	0.45 **	0.37 *	0.45 *	0.43 **	0.55 ***	0.19	0.58 ***	0.52 ***	−0.02
TO	0.39 *	0.41 **	0.38 *	0.44 **	0.47 **	0.53 ***	0.65 ***	0.26	0.46 **	0.13
FC	0.29	0.30	0.38 *	0.25	0.19	0.29	0.37 *	0.36 *	0.27	−0.03
FR	0.39 *	0.28	0.17	0.43 **	0.25	0.40 *	0.55 ***	0.11	0.32 *	0.36 *
ER	0.25	0.19	−0.00	0.35 *	0.18	0.27	0.22	−0.02	0.17	0.35 *
+/−	−0.04	0.02	0.00	−0.04	0.02	0.00	−0.10	−0.15	0.11	0.14
Possessions	0.25	0.35 *	0.35 *	0.33 *	0.05	0.13	0.16	0.45 **	0.19	−0.02
PPP	0.18	0.16	0.00	0.46 **	0.16	0.33 *	0.26	−0.08	0.15	0.33 *
eFG (%)	0.18	0.15	0.07	0.37 *	0.13	0.26	0.05	−0.00	0.12	0.19
ORB (%)	−0.28	−0.23	−0.28	−0.16	0.00	−0.19	−0.19	−0.35 *	−0.16	−0.08
DRB (%)	−0.08	0.02	−0.02	0.02	0.12	0.08	0.21	−0.26	−0.10	0.17
TS (%)	0.18	0.11	−0.04	0.33 *	0.11	0.20	0.08	−0.05	0.14	0.24
PU (%)	0.13	0.45	0.03	0.42 **	0.26	0.44 **	0.40 *	−0.12	0.24	0.21
A/TO	0.20	0.26	0.09	−0.03	0.00	0.06	0.03	0.30	0.31	0.02
Partial correlation (controlling played time variable)										
Points	−0.17	−0.12	−0.12	0.34 *	0.15	0.31	0.08	−0.54 **	0.01	0.14
1FG (A)	−0.10	−0.11	−0.33	−0.29	−0.18	−0.33	0.15	−0.41	−0.07	0.57 *
2FG (A)	−0.10	0.09	0.09	0.44 *	0.28	0.46 **	0.27	−0.32	0.25	0.05
3FG (A)	−0.03	0.72	−0.04	−0.03	0.12	0.18	0.03	0.04	0.25	0.11
OR	−0.13	0.88	−0.16	0.05	0.04	−0.11	0.01	−0.17	−0.01	−0.01
DR	−0.08	0.46	−0.01	0.19	0.13	0.13	0.27	−0.20	−0.03	−20
TR	−0.12	0.69	−0.07	0.14	0.08	−0.02	0.21	−0.24	−0.02	0.15
Assists	0.31	0.28	0.10	−0.07	0.06	0.12	0.06	0.26	0.33 *	0.02
Steals	0.26	0.22	0.22	0.23	0.37 *	0.48 **	0.02	0.40 *	0.39 *	−0.08
TO	0.17	0.34 *	0.32	0.37 *	0.44 **	0.49 **	0.59 ***	0.12	0.41 *	0.08
FC	0.14	0.24	0.34 *	0.18	0.16	0.25	0.28	0.33 *	0.21	−0.08
FR	−0.10	−0.08	−0.06	0.16	0.15	0.29	0.41 *	−0.43 **	0.12	0.33 *
ER	−0.15	−0.12	−0.22	0.12	0.08	0.16	0.05	−0.52 **	−0.02	0.31
+/−	0.22	0.14	0.06	0.06	0.05	0.05	−0.03	−0.07	0.19	0.16
Possessions	0.04	−0.15	0.10	−0.18	−0.17	−0.13	0.04	−0.08	−0.18	−0.07
PPP	−0.22	−0.11	−0.18	0.31	0.08	0.24	0.13	−0.52 ***	−0.02	0.30
eFG (%)	−0.07	−0.09	−0.09	0.21	0.05	0.17	−0.09	−0.38 *	−0.04	0.16
ORB (%)	−0.11	−0.03	−0.17	0.07	0.09	−0.09	−0.08	−0.17	−0.01	−0.04
DRB (%)	−0.06	−13	0.050	0.14	0.16	0.13	0.28	−0.23	−0.05	0.18
TS (%)	−0.14	−0.17	−0.22	0.14	0.03	0.10	−0.07	−0.47 **	−0.03	0.20
PU (%)	0.21	0.19	0.02	−0.18	−0.04	0.00	−0.04	0.25	0.27	0.18
AT/RT	−0.06	0.04	−0.03	0.42 **	0.24	0.42 *	0.36 *	−0.33 *	0.20	0.00

+/−: player balance; A/TO: assists–turnover ratio; AT: active time; COI: changes in inertia; DR: defensive rebounds; DRB: defensive rebound percentage; DSL: dynamic stress load; eFG: efficiency field goal percentage; ER: efficiency rating; FC: fouls committed; FG: field goals (made/attempted); FR: fouls received; Hi-COI: high-intensity changes in inertia; Hi-HI: high-intensity horizontal impacts; Hi-Landing: jumps’ high-intensity landing; Hi-PL: high-intensity player load; Hi-Takeoff: jumps’ high-intensity takeoff; OF: offensive rebounds; ORB: offensive rebound percentage; PL: player load; PP: number of players who participated; PPP: points per possession; TP: total of players available for playing; RT: recovery time; TR: total rebounds; USG: usage percentage; AU: arbitrary units; min: minutes. * *p* < 0.05, ** *p* < 0.01, *** *p* < 0.001; ^a^: Pearson’s correlation coefficients; ^β^: Spearman correlation coefficients.

## Data Availability

Data are contained within the article.

## References

[1] Montgomery P.G., Pyne D.B., Minahan C.L. (2010). The Physical and Physiological Demands of Basketball Training and Competition. Int. J. Sports Physiol. Perform..

[2] Fox J.L., Scanlan A.T., Stanton R. (2017). A Review of Player Monitoring Approaches in Basketball: Current Trends and Future Directions. J. Strength Cond. Res..

[3] Russell J.L., McLean B.D., Impellizzeri F.M., Strack D.S., Coutts A.J. (2021). Measuring Physical Demands in Basketball: An Explorative Systematic Review of Practices. Sports Med..

[4] Reina M., García-Rubio J., Pino-Ortega J., Ibáñez S.J. (2019). The Acceleration and Deceleration Profiles of U-18 Women’s Basketball Players during Competitive Matches. Sports.

[5] Reina M., Mancha-Triguero D., Ibáñez S.J. (2022). Monitoring of a Competitive Microcycle in Professional Women’s Basketball through Inertial Devices. Rev. Int. Med. Cienc. Act. Fis. Deporte.

[6] Espasa-Labrador J., Calleja-González J., Montalvo A.M., Fort-Vanmeerhaeghe A. (2023). External Load Monitoring in Female Basketball: A Systematic Review. J. Hum. Kinet..

[7] Petway A.J., Freitas T.T., Calleja-González J., Leal D.M., Alcaraz P.E. (2020). Training Load and Match-Play Demands in Basketball Based on Competition Level: A Systematic Review. PLoS ONE.

[8] Espasa-Labrador J., Fort-Vanmeerhaeghe A., Montalvo A.M., Carrasco-Marginet M., Irurtia A., Calleja-González J. (2023). Monitoring Internal Load in Women’s Basketball via Subjective and Device-Based Methods: A Systematic Review. Sensors.

[9] McLaren S.J., Macpherson T.W., Coutts A.J., Hurst C., Spears I.R., Weston M. (2018). The Relationships between Internal and External Measures of Training Load and Intensity in Team Sports: A Meta-Analysis. Sports Med..

[10] Nicolella D.P., Torres-Ronda L., Saylor K.J., Schelling X. (2018). Validity and Reliability of an Accelerometer-Based Player Tracking Device. PLoS ONE.

[11] Robertson S., Duthie G.M., Ball K., Spencer B., Serpiello F.R., Haycraft J., Evans N., Billingham J., Aughey R.J. (2023). Challenges and Considerations in Determining the Quality of Electronic Performance & Tracking Systems for Team Sports. Front. Sports Act. Living.

[12] Salazar H., Ujakovic F., Plesa J., Lorenzo A., Alonso-Pérez-Chao E. (2024). Do Elite Basketball Players Maintain Peak External Demands throughout the Entire Game?. Sensors.

[13] Straeten M., Rajai P., Ahamed M.J. (2019). Method and Implementation of Micro Inertial Measurement Unit (IMU) in Sensing Basketball Dynamics. Sens. Actuators A Phys..

[14] García-de-Alcaraz A., Rico-González M., Pino-Ortega J. (2022). Criterion Validity and Reliability of a New Algorithm to Detect Jump Performance in Women’s Volleyball Players. Proc. Inst. Mech. Eng. Part P J. Sports Eng. Technol..

[15] Avilés R., Brito de Souza D., Pino-Ortega J., Castellano J. (2023). Agreement, Accuracy, and Reliability of a New Algorithm for the Detection of Change of Direction Angle Based on Integrating Inertial Data from Inertial Sensors. Algorithms.

[16] Oliva-Lozano J.M., Chmura P., Granero-Gil P., Muyor J.M. (2023). Using Microtechnology and the Fourier Transform for the Analysis of Effective Activity Time in Professional Soccer. J. Strength Cond. Res..

[17] García F., Fernández D., Martín L. (2022). Relationship Between Game Load and Player’s Performance in Professional Basketball. Int. J. Sports Physiol. Perform..

[18] Fox J.L., Stanton R., O’Grady C.J., Teramoto M., Sargent C., Scanlan A.T. (2022). Are Acute Player Workloads Associated with In-Game Performance in Basketball?. Biol. Sport.

[19] Vázquez-Guerrero J., Casals M., Corral-López J., Sampaio J. (2020). Higher Training Workloads Do Not Correspond to the Best Performances of Elite Basketball Players. Res. Sports Med..

[20] Leicht A., Gomez M., Woods C. (2017). Team Performance Indicators Explain Outcome during Women’s Basketball Matches at the Olympic Games. Sports.

[21] NBA Advanced Stats Glossary. https://www.nba.com/stats/help/glossary.

[22] Bezerra M. (2023). Performance Analysis in Elite Basketball Differentiating Game Outcome and Gender. Eur. J. Hum. Mov..

[23] Oliva-Lozano J.M., Rojas-Valverde D., Gómez-Carmona C.D., Fortes V., Pino-Ortega J. (2021). Impact of Contextual Variables on the Representative External Load Profile of Spanish Professional Soccer Match-play: A Full Season Study. Eur. J. Sport Sci..

[24] McKay A.K.A., Stellingwerff T., Smith E.S., Martin D.T., Mujika I., Goosey-Tolfrey V.L., Sheppard J., Burke L.M. (2022). Defining Training and Performance Caliber: A Participant Classification Framework. Int. J. Sports Physiol. Perform..

[25] World Medical Association (2013). World Medical Association Declaration of Helsinki. JAMA.

[26] (2016). European Parliament and Council, Regulation.

[27] Gómez-Carmona C.D., Rojas-Valverde D., Rico-González M., Ibáñez S.J., Pino-Ortega J. (2021). What Is the Most Suitable Sampling Frequency to Register Accelerometry-Based Workload? A Case Study in Soccer. Proc. Inst. Mech. Eng. Part P J. Sports Eng. Technol..

[28] Gómez-Carmona C.D., Bastida-Castillo A., González-Custodio A., Olcina G., Pino-Ortega J. (2020). Using an Inertial Device (WIMU PRO) to Quantify Neuromuscular Load in Running: Reliability, Convergent Validity, and Influence of Type of Surface and Device Location. J. Strength Cond. Res..

[29] Beato M., De Keijzer K.L., Carty B., Connor M. (2019). Monitoring Fatigue during Intermittent Exercise with Accelerometer-Derived Metrics. Front. Physiol..

[30] Reina M., Mancha-Triguero D., García-Santos D., García-Rubio J., Ibáñez S.J. (2019). Comparison of Three Methods of Quantifying the Training Load in Basketball. RICYDE Rev. Int. Cienc. Deporte.

[31] Hernández-Belmonte A., Bastida-Castillo A., Gómez-Carmona C.D., Pino-Ortega J. (2019). Validity and Reliability of an Inertial Device (WIMU PROTM) to Quantify Physical Activity Level through Steps Measurement. J. Sports Med. Phys. Fit..

[32] Lalanne C., Mesbah M. (2016). Measures of Association, Comparisons of Means and Proportions for Two Samples or More. Biostatistics and Computer-Based Analysis of Health Data using Stata.

[33] Cohen J. (2013). Statistical Power Analysis for the Behavioral Sciences.

[34] Jamovi Project. https://www.jamovi.org/.

[35] Gómez M.Á., Lorenzo A., Jiménez S., Navarro R.M., Sampaio J. (2015). Examining Choking in Basketball: Effects of Game Outcome and Situational Variables during Last 5 Minutes and Overtimes. Percept. Mot. Ski..

[36] García F., Vázquez-Guerrero J., Castellano J., Casals M., Schelling X. (2020). Differences in Physical Demands between Game Quarters and Playing Positions on Professional Basketball Players during Official Competition. J. Sports Sci. Med..

[37] Portes R., Navarro Barragán R.M., Calleja-González J., Gómez-Ruano M.Á., Jiménez Sáiz S.L. (2022). Physical Persistency across Game Quarters and during Consecutive Games in Elite Junior Basketball Players. Int. J. Environ. Res. Public Health.

[38] Alonso Pérez-Chao E., Gómez M.-Á., Lisboa P., Trapero J., Jiménez S.L., Lorenzo A. (2022). Fluctuations in External Peak Demands Across Quarters During Basketball Games. Front. Physiol..

[39] Oliva-Lozano J.M., Conte D., Fortes V., Muyor J.M. (2023). Exploring the Use of Player Load in Elite Soccer Players. Sports Health A Multidiscip. Approach.

[40] Reina M., García-Rubio J., Esteves P.T., Ibáñez S.J. (2020). How External Load of Youth Basketball Players Varies According to Playing Position, Game Period and Playing Time. Int. J. Perform. Anal. Sport.

[41] Delextrat A., Badiella A., Saavedra V., Matthew D., Schelling X., Torres-Ronda L. (2015). Match Activity Demands of Elite Spanish Female Basketball Players by Playing Position. Int. J. Perform. Anal. Sport.

[42] Vázquez-Guerrero J., Jones B., Fernández-Valdés B., Moras G., Reche X., Sampaio J. (2019). Physical Demands of Elite Basketball during an Official U18 International Tournament. J. Sports Sci..

[43] Novillo Á., Cordón-Carmona A., García-Aliaga A., Roman I.R., del Campo R.L., Resta R., Buldú J.M. (2024). Analysis of Player Speed and Angle toward the Ball in Soccer. Sci. Rep..

[44] Gutiérrez-Vargas R., Pino-Ortega J., Ugalde Ramírez A., Sánchez-Ureña B., Blanco-Romero L., Trejos-Montoya J., Gutiérrez-Vargas J.C., Rojas-Valverde D. (2022). Physical and Physiological Demands According to Gender, Playing Positions, and Match Outcomes in Youth Basketball Players. RICYDE Rev. Int. Cienc. Deporte.

[45] Oliveira-Da-Silva L., Sedano-Campo S., Redondo-Castán J.C. (2013). Características Del Esfuerzo En Competición En Jugadoras de Baloncesto de Élite Durante Las Fases Finales de La Euroliga y El Campeonato Del Mundo. RICYDE Rev. Int. Cienc. Deporte.

[46] Delextrat A., Trochym E., Calleja-González J. (2012). Effect of a Typical In-Season Week on Strength Jump and Sprint Performances in National-Level Female Basketball Players. J. Sports Med. Phys. Fit..

[47] Konefał M., Chmura J., Zacharko M., Zając T., Chmura P. (2023). The Relationship among Acceleration, Deceleration and Changes of Direction in Repeated Small Sided Games. J. Hum. Kinet..

[48] Clemente F.M., González-Fernández F.T., García-Delgado G., Silva R., Silva A.F., Nobari H., Falces-Prieto M. (2022). Leg Dominance and Performance in Change of Directions Tests in Young Soccer Players. Sci. Rep..

[49] Gasperi L., Sansone P., Gómez-Ruano M.-Á., Lukonaitienė I., Conte D. (2023). Female Basketball Game Performance Is Influenced by Menstrual Cycle Phase, Age, Perceived Demands and Game-Related Contextual Factors. J. Sports Sci..

[50] Brown F.S.A., Fields J.B., Jagim A.R., Baker R.E., Jones M.T. (2024). Analysis of In-Season External Load and Sport Performance in Women’s Collegiate Basketball. J. Strength Cond. Res..

[51] Nin D.Z., Lam W.K., Kong P.W. (2016). Effect of Body Mass and Midsole Hardness on Kinetic and Perceptual Variables during Basketball Landing Manoeuvres. J. Sports Sci..

